# ‘I want food to be tasty and healthy’: school-children’s experiences with nutrition education and messaging

**DOI:** 10.1093/heapro/daaf151

**Published:** 2025-10-01

**Authors:** Fiona Quigley, Louise Lynch, Ruth Price, Lynsey Hollywood, Alison M Gallagher, Elaine Mooney, Amanda McCloat, S Anne Moorhead

**Affiliations:** Institute for Nursing and Health Research, School of Communication and Media, Ulster University, York Street, Belfast, BT15 1ED, Northern Ireland; School of Communication and Media, Ulster University, York Street, Belfast, BT15 1ED, Northern Ireland; Nutrition Innovation Centre for Food and Health, School of Biomedical Sciences, Ulster University, Coleraine BT52 1SA, Northern Ireland; Department of Hospitality Tourism and Events Management, Ulster University Business School, Ulster University, York Street, Belfast, BT15 1ED, Northern Ireland; Nutrition Innovation Centre for Food and Health, School of Biomedical Sciences, Ulster University, Coleraine BT52 1SA, Northern Ireland; National Centre of Excellence for Home Economics, School of Home Economics, ATU St Angelas, Lough Gill, Co. Sligo F91 C634, Ireland; National Centre of Excellence for Home Economics, School of Home Economics, ATU St Angelas, Lough Gill, Co. Sligo F91 C634, Ireland; Institute for Nursing and Health Research, School of Communication and Media, Ulster University, York Street, Belfast, BT15 1ED, Northern Ireland

**Keywords:** food messaging, nutrition education, children, adolescents, healthy eating

## Abstract

Ensuring that children and adolescents receive adequate nutrition is a cornerstone of public health globally, supporting their growth, development, and long-term well-being, but not enough is known about children and adolescents’ perspectives on nutrition. The aim of this study was to obtain the perceptions and attitudes of school-aged children and adolescents (4–18 years) on existing models and approaches to food education and food messages. Data were collected using friendship paired interviews and focus groups from the children and adolescents (*n* = 70) within Northern Ireland (NI) and Republic of Ireland (ROI) in seven primary and six post-primary schools, which were recruited to reflect different groups in terms of gender, age, region and rural/urban. Data were analysed using reflexive thematic analysis (RTA) with NVivo supporting the coding and analysis. Three themes were identified, (i) ‘Impactful messaging’; (ii) ‘Guidance and support’, and (iii) ‘Improving messaging and education’. This study found that food messaging and delivery needs to be age-appropriate, consistent and accurate from multiple sources to cut through the ‘noise’ of less healthy food messages. Children and adolescents want increased voice and agency in their food education, as they know what works for them, such as opportunities with peers and social media. By using co-design methodologies, food education can be better aligned with their needs and interests. Striking the right balance between ‘tasty’ and ‘healthy’ is a clear recommendation from children and adolescents to rethink their involvement in food education.

Contribution to Health Promotion

- Reports lived experience insights from children and adolescents on food messaging and nutrition education.
- Uses an ecological lens to explore psychosocial, physiological, and environmental influences on food engagement.
- Highlights how children and adolescents prefer to engage with nutrition education across life stages and contexts.
- Connects findings to global, multidisciplinary discussions on children and adolescent health and food environments.
- Offers actionable recommendations for policy, practice, and future research on food messaging for children and adolescents.

## INTODUCTION

Ensuring that children and adolescents receive adequate nutrition is a cornerstone of public health globally, supporting their growth, development, and long-term well-being (WHO 2023). However, persistent gaps remain between dietary recommendations and actual food and nutrient intakes in many countries. In both the UK and USA, children and adolescents often fail to meet dietary guidelines; the National Diet and Nutrition Survey (NDNS) reported free sugar intake in 4–18-year-olds exceeds the 5% recommendation (Public Health England 2020), and National Health and Nutrition Examination Survey (NHANES) data show many young people lack adequate nutrient intake [Centers for Disease Control and Prevention (CDC) 2021]. These dietary patterns contribute to both nutrient deficiencies and overnutrition, undermining the health of younger generations and increase the risk of chronic disease into adulthood (Alves and Alves 2024; Ghosh *et al*. 2024).

Over the past four decades, the food environment, on which children and adolescents rely, has shifted dramatically due to technological advancements, globalization, and societal changes (Kemmer 2000, Varela *et al*. 2024). Central to the food environment is ‘food messaging’, a term with no agreed definition, that describes a form of communication that carries meaning related to food/beverages, food activities, diet/nutrients, systems and technologies that are intentionally made and can transmit knowledge that shapes food practices, preferences, standards, ideologies and norms (Ruxton *et al*. 2023, Cuykx *et al*. 2024). Food messages can be delivered through any medium, typically including verbal, image-based or text, across domains including public health campaigns, nutrition education, advertising and social media and by authors including institutions, artifical intelligence (AI) or human. Changes in food messaging, particularly in Western countries, have occurred alongside the food environment shifts and are often created with commercial intent (Cuykx *et al*. 2024; Hawkins and Rundle 2024). In the 1970s and 1980s, food messaging was primarily disseminated through traditional media (television, radio, and print) with limited opportunities for advertisements (Aspray *et al*. 2014). In 2025, children and adolescents navigate a digital ecosystem where personalized advertising and algorithm-driven content on social media exposes them to near-constant targeted food marketing (Nieto *et al*. 2023; Maksi *et al*. 2024). Digital advertising differs from traditional forms (Harris *et al*. 2025), employing sophisticated tactics, including gamification and influencer marketing, to promote less healthy food choices, often overshadowing public health efforts to encourage healthier eating (Tatlow-Golden and Garde 2020; Qutteina *et al*. 2021; Bozzola *et al*. 2022). Meanwhile, family food practices, which traditionally transferred family food messages, supported healthier eating and skills development, have shifted from shared, home-cooked meals towards greater reliance on fast food and ready-to-eat meals, facilitated by delivery apps and the widespread availability of cheap, nutrient-poor options (McKinsey & Company 2021; Lavelle 2023). These changes have reduced the impact of nutritional guidance within families and communities, leaving children and adolescents more vulnerable to external and often commercial food messaging (Boyland *et al*. 2024; Harris *et al*. 2025).

Children and adolescents are active participants in the food environment; creating, sharing, and consuming food-related content online, often engaging with trends that promote less healthier foods (Boyland *et al*. 2024). Social media has fundamentally changed how children and adolescents engage with food-related content. Platforms such as Instagram, TikTok, and YouTube are now central hubs where children and adolescents encounter food messaging that often prioritizes entertainment and consumption over education and health (Bozzola *et al*. 2022). These platforms offer opportunities for innovative health communication but also risks when the content is created by individuals or organizations with vested commercial interests. There is potential for exposure to misinformation from those who can lack adequate nutrition qualifications (Bozzola *et al*. 2022, Meléndez-Illanes *et al* 2022). Traditional public health tools that promote healthier food messaging, such as the UK’s ‘Eatwell Guide’ (National Health Service 2022), the ‘Food Pyramid’ in Ireland (Department of Health 2016), and the USA’s ‘MyPlate’ (Center for Nutrition Policy and Promotion 2023), might not have evolved in parallel to the rapidly changing food environment on social media. Despite efforts to improve school and community food environments, children and adolescents continue to face conflicting messages in educational settings, often with access to both nutritious and less healthy options (Maher *et al*. 2020, Hawkings and Rundle 2024). Educators and policymakers need to rethink food messaging for children and adolescents, including digital-first strategies, interactive tools, and influencer partnerships to make healthy eating messages more relevant and appealing, while incorporating children and young people’s perspectives to ensure resonance (Public Health England 2020; Tatlow-Golden and Garde 2020).

Understanding children and adolescents’ autonomy and ability to make food choices is essential when considering how they experience food messaging. Supporting children and adolescents in developing self-regulation skills and navigating the food environment is challenging, as they learn from, and depend on, adults at home and in school to create healthier food environments (Varela *et al.* 2024 ) and provide nutrition education. Early feeding practices significantly shape children's eating behaviours (Slaughter and Bryant 2004; Coulthard *et al*. 2010), with instrumental or restrictive feeding approaches potentially leading to unintended consequences, including a higher risk of disordered eating in adolescence (Herle *et al*. 2020; Powell *et al*. 2023). Additionally, Ruxton *et al*. (2023) describe the ‘noisy’ messaging environment, where children navigate competing healthier versus less healthy food messages and suggest focusing on fewer, easier to implement and clearer messages that communicate straightforward health benefits.

Addressing the combination of challenges requires more than just messaging strategies, and the inclusion of children and adolescents’ perspectives. To date, research on food messaging and nutrition education has prioritized adult-driven perspectives, focusing on expert views rather than those of children and adolescents themselves (Thomas *et al*. 2003, Russell *et al*. 2023, Wright-Pedersen *et al*. 2024). Involving children and adolescents in designing food messaging and nutrition education is best practice and enacts their right to participate in decisions affecting their lives, including health and nutrition, as asserted by the United Nations ‘Convention on the Rights of the Child’ (United Nations 1989). Participatory approaches also have demonstrated potential for improving the relevance, appeal, and effectiveness of health communication strategies (WHO 2016). Addressing this gap by examining children’s and adolescents’ experiences with nutrition education and food messaging can centre their voices and support insight into how they interpret, value, and act on food messages (van der Bend *et al*. 2024). By improving understanding on how children and young people navigate healthy food messaging versus less healthy food messaging, researcher, policy makers, and practitioners can learn how to better support positive relationships with food and improve nutrition and health outcomes for children and adolescents.

### Study aim

The aim of this study was to obtain the perceptions and attitudes of school-aged children and adolescents (4 −18 years) on existing models and approaches to nutrition education and food messages.

## METHODS

### Overall project scope

This study was part of a larger three-stage research project (*n* = 396) funded by Safefood Ireland (February 2023 to October 2024), titled ‘Food messaging to children and adolescents—What works?’ This included a project advisory group (*n* = 8), representing stakeholder groups.

### Research design

This qualitative study used reflexive thematic analysis (RTA) (Braun and Clarke 2021) to explore children’s and adolescents’ perspectives on food messaging and nutrition education. To ensure methodological congruence in reporting, this research used both the Qualitative Design Reporting Standards (APA 2024) and RTA reporting recommendations by Braun and Clarke (2024), including considerations of ‘knowingness, positionality, reflexivity, and information power’ (Braun and Clarke 2021, 2024). Given the inclusion of children and adolescents, a partnership approach was adopted, with participation strategies embedded within the design to ensure developmental appropriateness and comfort.

### Reflexivity

Throughout data collection and analysis, authors discussed and acknowledged their unique perspectives and checked for shared understandings of terms, research goals, and values. In particular, the team reflected on beliefs held about children and young people’s abilities to contribute to research and how this might inform analysis and how information power was generated. Acting as ‘critical friends’ (Braun and Clarke 2024), we discussed how to approach data analysis with integrity, ensuring that youth voice and experience were centred. Researcher reflexivity was maintained throughout the project, using field notes, memos and discussions with the research team. The inclusion of rich descriptions of data in reporting also supported trustworthiness that the findings, demonstrating that findings were embedded in the data collected (Golafshani 2003; Lambert and Loiselle 2008).

### Study participants

This research took place on the island of Ireland with children and adolescents of school going age (4–18 years), which includes the independent state of ‘Republic of Ireland’ (ROI) and a region of the United Kingdom operating under a devolved government, ‘Northern Ireland’ (NI) (Hayward 2017). Participant characteristics are available in Table 1.

**Table 1.** Participant characteristics for the friendship pairs (*n* = 24) and focus groups (*n* = 46) .

	Friendship pairs	Focus groups
Total participants	*n* = 24	*n* = 46
Schools	7	6
Type of school	6 Co-educational, 1 all-girls	4 Co-educational, 1 all-girls
Number of discussion groups	12 (2 per pair)	6 (7/8 per group)
Mean (range) length of recording	17 (15–30) min	42 (40–60) min
Location by counties in NI and ROI	Derry (1 pair), Down (1 pair), Antrim (2 pairs), Donegal (2 pairs), Sligo (2 pairs), Cork (2 pairs, all -girls), and Dublin (2 pairs)	Antrim, Armagh, Sligo, Cork (all-girls), and Dublin
Gender (male/female)	10/14	Male—*n* = 16/30
Ethnicity	White Irish/British (*n* = 24)	White Irish/British (*n* = 44) Other white background (*n* = 2)
Age (years)	4—*n* = 1 5—*n* = 1 6—*n* = 4 7—*n* = 2 8—*n* = 5 9—*n* = 3 10—*n* = 1 11—*n* = 4 12—*n* = 3	12—*n* = 6 13—*n* = 7 14—*n* = 6 15—*n* = 15 16—*n* = 4 17—*n* = 8
Mean age (years)	8 (SD 2.4)	15 (SD 1.6)

### Participant recruitment

School principals in primary and post-primary schools across NI and ROI were emailed a study information pack, which included age-appropriate participant information sheets, and later contacted by phone to invite their school to participate. Participating schools then forwarded the recruitment e-mail pack to parents. This study employed both ‘purposive sampling and snowball sampling’ strategies for the recruitment of principals, children, and adolescents (Clark *et al*. 2021). Student participants were purposively selected for their school level: (i) ‘Primary’ (4–12 years) and (ii) ‘Post-primary’ (12–18 years). Snowball sampling was used as an important relational approach to safely recruit children and adolescents (Barbour and Barbour 2003; Ghaljaie Naderifar and Goli 2017). Those who wanted to participate were asked to provide parental consent and child/adolescent assent. All participants received a £20 Amazon voucher token of appreciation, and each school received £100 voucher.

#### Ethical considerations

This study was approved by Ulster University Research Ethics Committee (REC/23/0019) and data management adhered to the EU and UK General Data Protection Regulation (2018) and UK Data Protection Act (2018). Safeguarding practices as per school policies were followed. The researcher (FQ) was vetted by the schools and local authorities prior to data collection and conducted follow-ups and duty-of-care check-ins with schools. Participants and schools reported positive experiences.

### Data collection

Data collection took place in schools on the island of Ireland from June 2023 to November 2023. Important features when conducting qualitative research included attention to rapport, positive regard and active listening (Prior 2018). ‘Friendship pairs’, where two children self-select to participate together, were used for primary school children (McGuffin *et al*. 2015), and ‘focus groups’ (6–8 participants) were chosen for post-primary school adolescents, as developmentally appropriate methods to discuss models and approaches on food education and food messages (Creswell and Creswell 2017; Clark *et al*. 2021). No participants withdrew prior or during data collection. A classroom assistant, teacher or principal was present in all sessions for safeguarding purposes but did not take part in the discussions. The goal of achieving sufficient richness and depth of data, ‘information power’, was attained in this study (Braun and Clarke 2021, 2023).

#### Interview and focus group guides

Literature reviewing identified food messaging models and nutrition education approaches that informed the friendship pair/focus group guide. Two commonly used food models in school curricula on the island of Ireland, namely the ‘Eatwell guide’ (NI) (Multimedia file 2) and the ‘Food Pyramid’ (ROI)) (Multimedia file 3), were included. Questions for friendship pair/focus group guide were also informed using a bioecological perspective of food messaging (Bronfenbrenner 1995), by reflecting on how the child/adolescent receives different food messages across different contexts. This combination of literature and models led to an effective and age-appropriate friendship pairs/focus group guide that supported children and adolescents discussions on their food environment and food messaging (Table 2).

**Table 2.** Sample questions for friendship pair and focus group topic guide.

Friendship pairs with children	Focus groups with adolescents
Have you seen any of these pictures before? Where did you see them?	Have you encountered these examples before? If so, where?
Do you think it's important to eat different foods every day? Why or why not?	What are your initial thoughts or impressions about the examples shown?
How can these pictures help us understand which foods are good for our bodies?	Do you find these messages helpful in understanding nutrition? Why or why not?
Do you like to eat the foods shown in the pictures? Why or why not?	Do you feel motivated to follow the recommendations in these examples? Why or why not?
Is there anything about these pictures that you find hard to understand?	Do you find anything confusing or unclear?
Have you heard anything about these foods in school or from other places? Can you tell me about it?	Have you received any additional nutrition education or food messaging outside of these examples? If so, please share your experiences.
How do you think we can make learning about food fun and interesting for you?	How do you think food messaging could be improved to better resonate with young people?

Using age-appropriate language, engaging media and appropriate methods ensured a safe interactional space for children and adolescents. As a final check, the friendship pair/focus group guide was further refined by the project advisory group who suggested using two popular food messaging campaigns from NI and ROI: (i) Eat Them to Defeat Them (NI) (VegPower 2022) (see https://www.youtube.com/watch?v=C68TQ4uPcgg) and (ii) the Food Dudes campaign (ROI) (Morrill *et al*. 2016) (see https://www.youtube.com/watch?v=wxe8dNP8nf8&t) as they might help to spark discussion.

#### Procedures

Data collection with primary participants took place in five NI schools (urban *n* = 2, and rural *n* = 3) and eight schools in the ROI (urban *n* = 4, and rural *n* = 4). For post-primary participants, focus groups took place in two NI schools (1 urban and 1 rural) and four schools in ROI (2 urban and 2 rural). Data collection took place in suitable school meeting rooms and was audio recorded. Participants were welcomed, consent confirmed, and procedures explained. All participants were given visual copies of the food education models and asked for their opinions. For friendship pairs, a short ice breaker and fun activity supported rapport development with the researcher. Once the children were notably comfortable, data collection began. Using pictures of the food models, children discussed their thoughts with each other and the researcher. At post-primary level, questions were posed to the group, with individual responses generating group discussion. Participants were thanked and debriefed, and staffs were informed that data collection had ended.

### Data analysis

Transcription work was completed verbatim, and uploaded to NVivo (Version 14, QSR International Pty Ltd, 2023). The researcher (FQ) employed RTA methods, taking an inductive approach towards data analysis (Braun and Clarke 2021). This method reflects a theoretically flexible interpretivist approach that can support the analysis of children and adolescents’ perspectives on food messaging and nutrition education. During analysis the researcher remained open to the participant responses and explored data for meaning (Braun and Clarke 2023). Subjectivity is a valued and key part of the analysis, keeping researcher ‘positionality’ in mind as patterns or codes in the data are identified. The researcher (F.Q.) coded the data and used semantic and latent coding to identify patterns from the participants’ experiences following the six-stage process (Braun and Clarke 2021). Field notes were re-read, and a reflective diary supported reflexivity and tracked the first author’s subjectivity. Coding was discussed with other research team members (L.L. and A.M.), leading to refinements. Final themes were developed from and through coding (Braun and Clarke 2024) and reviewed with authors (L.L. and A.M.). Regarding data handling, primary and post-primary data sets were analysed separately, then compared for convergence and divergence, including age-related needs, gender and urban versus rural school location.

## RESULTS

**Figure 1.**
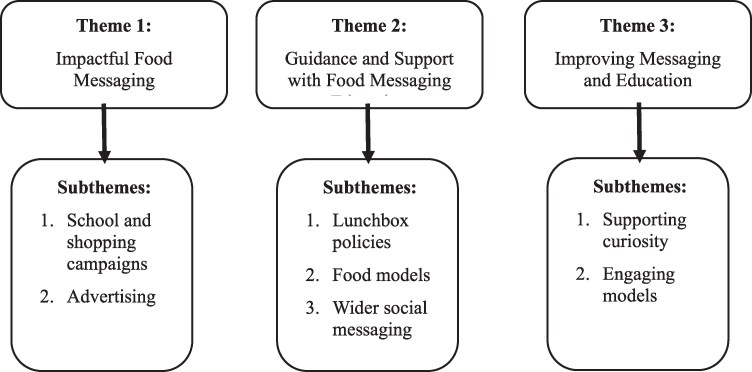
Key themes from the friendship pairs and focus group thematic analysis.

This section presents the combined analysis for friendship pairs (*n* = 24) and focus groups (*n* = 46). Differences between primary and post-primary datasets are highlighted throughout, as are findings in regard to reported age-related needs. Three overarching themes were generated from the friendship pairs and focus groups (i) ‘Impactful messaging’, (ii) ‘Guidance and monitoring’ and (iii) ‘Improving messaging and nutrition education’ (Fig. 1).

During analysis, no notable or novel gender differences regarding participant opinions on food messaging and models were identified. Similarly, no notable differences were found between children and young people in rural or urban settings, likely due to Ireland’s geographical context of the island of Ireland, where urban centres are small and rural areas are less isolated. It was also observed that primary school children typically had less access to social media, with access increasing into late primary and throughout post-primary.

### Theme one: impactful food messaging

#### School and shopping campaigns

Primary school participants recalled food messaging via school campaigns for vegetables, which were accompanied with prizes and rewards, ‘It was in the cinema too, there was lots of talk about the prizes and stuff, but I didn’t think it was that good’ (Male participant, 9 years) and many participants did not experience these campaigns as impactful: ‘I don’t really remember them [the campaign], but I think I saw some posters in our canteen’ (Female participant, 9 years). Participants at post-primary level also agreed with their primary counterparts, elaborating on how well-known supermarkets were currently using prizes and rewards as part of vegetable messaging campaigns, ‘There used to be something in [supermarket] that you could collect Smurfs for the amount of vegetables you buy, if you spent, I think, €10 on veg, you'd get a little character and you'd have to build the collection’ (Female participant, 17 years). For some, they focused on getting the rewards and prizes, and participation did not necessarily lead to eating more vegetables in the longer term, but some did report opportunities for exposure to new tastes, ‘I never tasted cucumber before, so I got to try it and thought it was ok’ (Female participant, 13 years). Post-primary school participants reflected on these campaigns and reported that just tasting vegetables was not enough to encourage eating more or a wider variety of vegetables. They would have liked to learn more about how to use the vegetables in recipes, demonstrating the age-related needs at post-primary level for skills development, ‘…if they’d showed us how to use them in dinners that could have been better’ (Female participant, 15 years). Post-primary participants described the influential role of peers who could play a supportive role or could cause challenges. Observing peers responding negatively to vegetables could influence others either way, ‘Sometimes others just didn’t even try the vegetables—like the broccoli and that could put you off but same goes if they did like them—you might try it too’ (Male participant, 14 years).

#### Advertisements

Post-primary participants recalled food advertisements and social media content that contained frequent references to food, ‘I remember ones with funny voices, the two policemen talking like children in the car was really funny’ (Male participant, 15 years). In general, participants mostly remembered posters at bus shelters on their way to school and in their local community and on social media applications such as ‘YouTube, Snapchat, Instagram and TikTok’, ‘I’d see people on YouTube eating different things, kind of promoting them and our pizza place is always on posters near us, leaflets through the door too’ (Male participant, 14 years). Food advertisements that were richly coloured and/or included offers, such as a certain percentage off, were more memorable, ‘McDs always have offers and its cheaper to buy a big box of nuggets than a smaller one’ (Male participant, 16 years). A smaller number of participants remembered seeing TV advertisements for large sweet and fast-food companies, recalling memorable music, comedy elements, rewards, and prize, ‘There is something about the [McDonalds] happy meals—all the little kids just want the toys’ (Female participant, 14 years). ‘TikTok’ was the most mentioned social media application, mainly by older participants (age > 12 years). Post-primary participants mentioned following ‘influencers’ who had recipes for food that they liked and that they thought were healthy, ‘I love pasta, so I go to TikTok for different pasta ideas, and they are quick and easy to make’ (Female participant, 15 years). Among older post-primary participants (age 14 + years), specific use of YouTube for ‘food challenges’ was mentioned, or watching people try out new local restaurants who sometimes issued the food challenges via social media, ‘We had a new Chinese [restaurant] and they were on YouTube with a competition to see who could eat the most of something—lots of people were talking about it’ (Male participant, 17 years).

### Theme two: guidance and support with food messaging

#### Lunch box policies

Primary students described how their schools tended to have a ‘lunch box policy’, which described the school’s vision for healthier eating and restricted the number of ‘treats’ they had in a week. No post-primary participants reported that their schools had lunchbox policies. Throughout this research, younger primary participants interpreted, due to their developmental capacity, that policy restrictions meant that a food was ‘bad’, ‘Sweets are bad for you, too much sugar hurts your teeth’ (Female participant, 5 years). This complemented findings with post-primary participants, who also reported understanding food messaging in ‘good and ‘bad’ terms, even when the messaging in schools itself did not use these terms, ‘You try to stick to more of the good stuff and less of the bad stuff’ (Male participant, 14 years). ‘Treats’ were reported as ‘bad food’, and included foods such as chocolate, biscuits, or crisps. Participants also reported varying interpretations by schools of what is acceptable regarding treats, with some school banning treats while others allowed one treat per week, ‘We can have a treat on a Friday, but you don’t have to—just if you wanted it’ (Female participant, 9 years).

#### Food models

All participants at primary and post-primary level, apart from the very youngest (age 4–5 years), had a familiarity with either the ‘Eatwell plate’ (NI) or the ‘Food Pyramid’ (ROI). Most participants reported some understanding of food proportions as laid out by the models, but it was not something they thought about or applied in their everyday eating, ‘I don’t really pay attention to that [portion sizes], I just eat when I’m hungry or what my mum gives me’ (Male participant, 14 years). Additionally, the younger the participant, the less likely they were to think about food model guidelines, as they relied on parents, mostly mothers, to prepare their lunches for school, and other meals in the home but family was also important for post-primary participants, ‘My Mum makes dinners like potatoes and carrots, but we get pizza on a Friday night too’ (Female participant, 13 years). Post-primary participants thought that cooked school lunches could be more ‘healthier’ and would be more likely to eat hot school meals if they had fresher and higher quality ingredients, ‘Our schools have hot dinners, but they look a bit beige, we could do with more vegetables’ (Male participant, 15 years). An additional finding regarded hydration, which was predominantly mentioned by post-primary participants, ‘I dance a lot and do a lot of sport … Mum is always reminding me to drink more water because I forget’ (Female participant, 15 years). Mothers were reported as more likely to monitor water intake and remind them about drinking water versus other more ‘sugary drinks’, as well as teaching about cooking, ‘I learn from my mum, she’d cook stuff and tell me ‘oh don’t put as much of that in it, only put a tiny bit of salt in … so that it is a wee bit healthier’ … you need that [guidance on healthy cooking]’ (Male participant, 15 years). Restricting sugary drinks was cited by participants as being one of the more difficult food messages to follow, ‘Compared to water it is just hard not to drink them, they taste much better—and you see them everywhere too’ (Male participant, 16 years).

#### Wider social messaging

While primary caregivers, typically mothers and fathers, were reported as communicating most of the food messaging in the home, grandparents were also mentioned as significant food messengers, ‘My granny would try to get me to eat vegetables too—I think my mum told her that to stop me eating sweets’ (Male participant, 10 years). Primary school participants stated that many of their grandfathers had vegetables gardens, and they enjoyed learning about how to grow different types of fruit and vegetables, ‘My Granda grows potatoes, carrots and peas. My favourites are the peas. And strawberries’ (Male participant, 9 years). Grandmothers were also reported as cooking on special occasions, and participants, particularly female participants, enjoyed learning to cook with their grandmothers, ‘We made a birthday cake once for my brother and I got to decorate it a bit’ (Female participant, 11 years).

Grandparents could communicate positive messages but could also undermine messages from school or from caregivers, ‘Granny gives me chocolate and crisps, so I know I’ll get them when I visit’ (Male participant, 11 years). Grandparents were also seen as a source of ‘treats’ or ‘bad’ foods, and the frequency of treats could sometimes cause tensions families due to contrary messaging, ‘It is what grandparents do but I know Mum sometimes tells her not to let me have a second (chocolate) bar’ (Male participant, 14 years). While participants communicated that they understood that too many treats were perceived as ‘bad’, they also noted that their grandparents were trying to be kind, and this behaviour was accepted as normal for them.

For participants who were involved in sport or clubs, such as Scouts or Girl Guides, most mentioned that they had at least one nutrition education talk from a coach, or someone brought into the club, ‘We have those posters [Food pyramid/Eatwell guide] up in our changing rooms and they remind us about the different foods to eat’ (Male participant, 13 years). Topics covered included how to eat before a game and keep well hydrated. Participants were told to eat ‘protein’ to give them energy but weren’t quite clear why. In general, few participants, regardless of age seemed to understand the role of different nutrients in the diet. Coaches or other experts also did not seem to cover post-game/meeting nutrition.

### Theme three: improving messaging and education

#### Supporting curiosity

Primary school participants predominantly showed interest in the production and economics of the food cycle. These participants reported interest in learning practical skills, wanting to learn how to grow fruit and vegetables and then how to cook them, ‘I go into the garden with Granda, and he explains how to get them to grow and then I help him to collect them. We had a lot of spuds this year’ (Male participant, 10 years). A few participants suggested that they would like to sell the fruit and vegetables they produced, either in the school tuck shop or community events in schools or through a community-based stall, ‘You could have a growing contest, like if you had a wee bed, you could divide into five plots and then they could have a growing contest and then you could eat the vegetables in school’ (Male participant, 8 years). Being exposed to a wider variety of food through a ‘taste the world day’ was a frequently mentioned idea, with some participants in post-primary schools stating that they were already doing this, ‘We tried food from different countries, like Greek and Spanish. I really liked the moussaka’ (Female participant, 14 years). In general, participants wanted more engaging and fun activities that included tasting and cooking new food, ‘I’d just like to be able to make tasty food with different recipes and not just talk about healthy all the time*’* (Female participant, 17 years). Adolescents also expressed curiosity and enthusiasm for visiting local food producers to see how different foods are made, ‘We visited a local bakery once and we saw how bread was made. I’d like to do more of that’ (Male participant, 15 years).

#### Engaging models

While the ‘Eatwell guide’ and ‘Food Pyramid’ guides are well known, many of the post-primary participants reported that there could be more practical support and learning around cooking healthy meals. There was a consensus that while they could understand and read food labels, which could support decision making, it would be much more beneficial to get ideas of sample meals, and recipes that were both ‘tasty and healthy’ but were also clearly labelled with all the nutrients, ‘Just more recipes with ideas of what you can do with the healthy ingredients and even a recipe which lists out all the nutrients so you know what is in it’ (Female participant, 14 years). Some participants expressed that ‘healthy’ food is often not that tasty and this impacted their relationship with understanding what ‘healthy’ food is. One participant reported the need for better food guidance that would be helpful to support younger people with understanding the meaning and nuance behind the guidance in the *Eatwell* guide, beyond ‘good’ and ‘bad’, to avoid developing restrictive or problematic eating behaviours, or for young people interpreting messaging as impossible and disengaging with healthy approaches entirely:

I think if people, seeing these messages, took them really seriously—for example, on dairy, on cheese, lower fat options, lower sugar options, some people might take that too literally at times and maybe take that out of their diet completely and it could just switch you off food. (Female participant, 14 years).

## DISCUSSION

### Developmentally appropriate communication

During this research, children and adolescents regularly referred to food in binary terms as being ‘good’ or ‘bad’, which might reflect how they interpret food messages that communicate food as ‘healthy’ or ‘unhealthy’ (Binder *et al*. 2021). This binary interpretation could result in children internalizing ‘healthy’ as ‘good’ and ‘unhealthy’ as ‘bad’, even into adolescence, suggesting the need for age-appropriate guidance and design of food messages and strategies that consider cognitive development (Papalia and Feldman 2011). Children and young people can also be supported with approaches that communicate food messages more effectively, improving their understanding of balance, benefits and impacts which can reduce food classification in traditional binary ways. For example, Wyllie Baxter and Kulczynski (2015) found that food messaging with children that uses ‘gain’ framing and affirmative messaging can support positive messages and intentions to adopt a target behaviour, and ‘loss’ framing or negation were most effective for trying to stop behaviours.

Engaging food messaging was described as using humour, music and colour—well-established marketing mechanisms (Dixon *et al*. 2017; Harris *et al*. 2025). This approach effectively markets fast foods to children and adolescents, altering their eating habits (Dixon *et al*. 2017; Smith *et al*. 2019) but has been difficult to replicate when marketing non-fast food, healthier new foods or public health messaging campaigns (Binder *et al*. 2021). Some healthy food promotion messaging has begun to use these methods successfully and this research found that the campaigns used in schools were somewhat useful and memorable. Recent findings show promise for frameworks that are developmentally designed to promote behaviour change, e.g. ‘Persuasive Strategies Presenting Healthy Foods to Children’, by Binder *et al*. (2021), but what leads to longer-term behaviour change is still a complicated topic.

Herle *et al*. (2020) describe eating behaviour as reflecting our responses to food cues in the environment and our relationship with food, with evidence that early life eating patterns are linked to later disordered eating behaviours. Findings from this research indicate that some young people can interpret eating guidelines more strictly, removing entire food groups from their diet that provide important macro or micronutrients. Some participants indicated that this could lead to problematic eating, and alongside other factors, contribute to the development of disordered eating and eating disorders (Herle *et al*. 2020). Participants also described fatigue when trying to understand food guidance, which could cause feelings of being unable to satisfy guidelines, leading to disengagement. Children and adolescents might need more practical support with eating guidelines, including examples on how a food model can fit into their daily life. In addition, as children and adolescents are dependent on their families for food, it is important to consider how food models align with families’ circumstances, which are impacted by the wider socio-economic circumstances and system factors, including access to high-quality, healthier foods (Stavitz 2024).

### Relationships and messaging

Children and adolescents in this research reported the different roles that their family members and friends played in food messaging and their nutrition education. The role of observational learning (Bandura *et al*. 1966) is widely accepted, and this research found that peers during adolescence are reported as playing an important role in influencing acceptability of foods (Collado-Soler *et al*. 2023; Holloway *et al*. 2023). Children and adolescents might be primed to observe how others respond to new foods as a way of learning about food, however, this could also be connected to food norms rooted in social relationships, identity and cultural values (Birch and Fisher 1998; Binder *et al*. 2021). Food neophobia can refer to how children can express reluctance, rejection or fear around trying new or unfamiliar foods (Biłek-Dratwa *et al*. 2022). There is a need to further explore the important role of peers at all ages in increasing exposure to new foods as a strategy in to support children or young people experiencing neophobia.

When discussing the role of families, participants in this study specifically noted their mothers and grandmothers, who scaffolded learning through guidance on ingredients, reinforcement over time, and monitoring eating for balance, all key activities in learning about food (Reddy and van Dam 2020, Perdew *et al*. 2021). Grandparents were found to be important role models (Criss *et al*. 2020), and while some of their ‘treat’ behaviours were viewed as problematic or causing the tension with parents, these were understood as expressions of affection or care. Grandparents were also described as important models for food preparation and production, connecting with findings by Jongenelis *et al*. (2021) and Criss *et al*. (2020). Future research can consider how to better involve and support grandparents in their roles (Beck *et al*. 2021), as intergenerational learning opportunities can be highly valued by both children and grandparents. Children and young people also reported receiving food messaging from their local sports clubs, predominantly through external speakers, with a focus on maintaining a healthy body for sports performance. A potential challenge was noted with speakers emphasizing single nutrients, such as protein promoted for performance, without explaining its specific role in a balance diet.

Adolescents discussed parasocial relationships with social media influencers (Hoffner and Bond 2022) and valued vicariously learning from their content, as a way to learn about food. Influencers also act as role models, helping young people access recipes for food they enjoy and potentially supporting skills development through cooking demonstrations. Social media can provide opportunities for adolescents to engage in food education and skills development in ways that align with their preferences. Social media is also an unregulated environment and this presents challenges as adolescents can interact with brands online in similar ways that they do with their friends, making them vulnerable to predatory marketing (Lutfeali *et al*. 2020). There is ample evidence to suggest that adolescents are developmentally vulnerable regarding digital marketing (Harris *et al*. 2025) and that there is inadequate policy to reduce their exposure (Sacks and Looi 2020). Food companies are frequently using tactics on social media to target adolescents with less healthier foods, including sugar-sweetened beverages (Théodore *et al*. 2021). A review by Bozzola *et al*. (2022) reported that there is an increased risk for children and adolescents on social media to targeted junk food marketing, pro-eating disorder content and sub-optimal quality information when they are seeking information about food, which can contribute to increased eating of unhealthier foods and problematic eating patterns. The task for policy and practice is to identify safe opportunities for activities online, and more research is needed that specifically looks at popular influencers or social media brands that provide adequate nutritional information or skills-based activities (Hollywood *et al*. 2022), and their role in supporting learning about nutrition education and promoting positive food messages.

### Children’s and adolescents’ food needs

Children and adolescents in this research noted that both school lunches and nutrition education, were often technically ‘healthy’ but not ‘tasty’. Factors such as limited time to eat or aspects of the social or school environment also impacted satisfaction with food. In research, food is recognized as important in self-regulatory behaviour (McCrickerd 2018) and is connected with cultural or community identity needs (eating food that their friends or family would eat), physiological needs (familiar textures and tastes), or psychological needs connected to their social or physiological needs (familiarity, expectation, safety, and consistency) (Slaughter and Bryant 2004; Reddy and van Dam 2020; Crawford *et al*. 2023; Varela *et al*. 2024). Children and adolescents wanting food to ‘taste’ good can be their way of communicating their multiple needs, highlighting that food messaging and education can consider the manifold purposes that food plays in their lives. Participants described their enjoyment and appreciation of food provided by family members, especially when they were cooking food together. Connecting with grandparents through gardening and food preparation activities had a positive effect on young people that went beyond mere exposure to a new taste or nutritional content (Criss *et al*. 2020, Jongenelis *et al*. 2021). These psychosocial connections to family and a sense of safety can help explain why children prefer familiar and predictable foods from their social environments (Slaughter and Bryant 2004; Varela *et al*. 2024). Connected with their physiological and psychological needs, children possibly more so than adolescents and especially in school settings, can express difficulty with unfamiliar foods, inconsistency in serving (menu or supplier changes) or presentation of foods in physical formats that they might not be used to or that they might have sensory issues with (e.g. mashed food). Food ‘fussiness’ might be connected to unmet food related needs but has also been reported as a potentially normative eating style during childhood (Herle *et al*. 2020). It is also possible that ‘fussiness’ can be linked to an adaptive evolutionary mechanism, where neophobia can appear as a typical developmental period of 2–6 years that serves to protect a person from ingesting potentially dangerous substances. However, avoidance behaviours can persist and extend further if children are not supported with food familiarization (Białek-Dratwa *et al*. 2022).

Whilst provision of school lunches can meet government policy and food model guidance, they do not necessarily meet all of children’s and adolescents’ needs, emphasizing the importance of inclusion and co-design of school meals and campaigns across domains (Wright-Pedersen *et al*. 2024).

### Food agency and life-stage considerations

Children and young people are not a homogenous demographic and experience constant changes and developmental challenges throughout childhood and youth (Best and Ban 2021). These findings support the need to consider the role of age in food messaging, with some research suggesting interventions at pre-school age (Mikkelsen *et al*. 2014) and others recommending school-age children who can be viewed as more flexible to change than adolescents (Perdew *et al*. 2021), or targeting early to mid-adolescence (11–16 years) (Brooks and Begley 2014; Calvert *et al*. 2019) when young people are becoming more independent. It is important to evaluate what works in reality, as adolescence is an established life-stage of social and identity development, and normative risk-taking, which might not be the most opportune developmental window to try and provide education on negative impacts of eating. Furthermore, with the impact of social media on body image (Bozzola *et al*. 2022) and the bodily changes during puberty that can result in self-consciousness, strategies need to tread carefully when discussing food in general to avoid contributing to the emergent increasing trend of eating disorders (Pastore *et al*. 2023). In addition, the ability of children and adolescents to make healthier food choices, referred to as ‘food agency’ (Wolfson and Leung 2020), is related to the degree to which their environments in schools, family and community settings facilitate this and how they move between these environments. Furthermore, gender differences have been observed in some studies indicating the need to consider this in future designs (Ajie and Chapman-Novakofski 2014, Calvert *et al*. 2019). Calvert *et al*. (2019) suggested that adolescents over 16 years might be more resistant to change due to natural maturation processes; however, youth is a developmental period in the lifespan of the individual that typically lasts from ages 10 to 30 years. There is a need to consider the life stage after adolescence—emerging adulthood (Arnett 2023), when young people continue identity development and have increased economic independence, as a potentially important opportunity for food messaging interventions to promote healthy eating and address the transgenerational impacts of eating and food choice. Developing messaging strategies from pre-school across childhood and youth, that are needs-based, developmentally appropriate and consider food agency, can support more effective and impactful food messaging.

### Limitations

Due to consideration of time and resource pressures for schools, participants were not asked to provide feedback on the themes.

### Recommendations for practice, policy, and research

- To review existing national guidelines and provide further information to children and adolescents on balanced and healthy dietary patterns, moving focus away from single foods, which can reduce children’s interpretation to a false dichotomy (good food/bad food).
- To embed food education throughout the school curriculum, ensuring relevancy to each subject with practical learning that leverages curiosity, rather than solely focus on formalized lessons in single subjects.
- Through co-design methodologies, to create and pilot age suitable food messages that support balance between development of food-related self-regulation skills and dietary restriction, which can reduce unintended outcomes connected with disordered eating patterns.
- To acknowledge that beyond nutrition, food has many different social and cultural functions for children, families and communities that need to be considered and included when devising food messaging strategies.
- To recognize that family-based food practices can be different to school-based practices and consider how best to devise messaging to be inclusive of both.
- To improve opportunities for intergenerational learning between students in schools and their family members.
- To identify safe opportunities for activities online to support children and adolescents to engage with healthier food messaging and be protected from predatory marketing.
- To conduct research to examine how influencers or social media brands can support adequate nutritional information or skills-based activities and promote positive food messages.
- Ensure children and adolescents from a range of backgrounds are included in the design of food messaging campaigns.

## CONCLUSION

Our food environment has shifted dramatically, where healthier food messages often compete with the allure of less healthy options for the attention of children and adolescents, who often have little food agency. This study concludes that food messaging and delivery needs to be age-appropriate, consistent and accurate from multiple sources to cut through the ‘noise’ of less healthy food messages. Children and adolescents are not passive observers; they want increased voice and agency in shaping their food education as they understand the obstacles they face in maintaining healthier eating habits, communicating clear ideas about what works for them including learning from peers and social media. Through co-design methodologies, including their perspectives and embracing food education that is social, practical, enjoyable, and aligned with their interests, healthier food messages may resonate better. Striking the right balance between ‘tasty’ and ‘healthy’ is not just a challenge, it is a clear recommendation from children and adolescents to inspire lasting change in how they relate to food and how adults in their lives can better meet their needs and contribute to their improved health outcomes.

## Data Availability

The data used in this study cannot be shared openly to protect participant privacy as participants did not consent to data availability.

## References

[daaf151-B1] Ajie WN, Chapman-Novakofski KM. Impact of computer-mediated, obesity-related nutrition education interventions for adolescents: a systematic review. J Adolesc Health 2014;54:631–45. 10.1016/j.jadohealth.2013.12.019 24534357

[daaf151-B2] Alves JGB, Alves LV. Early-life nutrition and adult-life outcomes. J Pediatr (Rio J) 2024;100:S4–9. 10.1016/j.jped.2023.08.007 37813343 PMC10960187

[daaf151-B3] American Psychological Association . *Qualitative Design Reporting Standards*, 2024. https://apastyle.apa.org/jars/qual-table-1.pdf (12 December 2024, date last accessed).

[daaf151-B4] Arnett JJ . Emerging Adulthood: the Winding Road from the Late Teens Through the Twenties. USA: Oxford University Press, 2023.

[daaf151-B5] Aspray W, Royer G, Ocepek MG et al Protecting children from obesity: a history of television and internet food advertising regulation in the United States. In: Formal and Informal Approaches to Food Policy. Cham: Springer International Publishing, 2014, 23–59.

[daaf151-B6] Bandura A, Grusec JE, Menlove FL. Observational learning as a function of symbolization and incentive set. Child Dev 1966;37:499. 10.2307/1126674 4165810

[daaf151-B7] Barbour RS, Barbour M. Evaluating and synthesizing qualitative research: the need to develop a distinctive approach. J Eval Clin Pract 2003;9:179–86. 10.1046/j.1365-2753.2003.00371.x 12787181

[daaf151-B8] Beck S, Criss S, Horhota M et al Intergenerational learning opportunities: a review of practices and outcomes. Educ Res Rev 2021;34:100421. 10.1016/j.edurev.2021.100421

[daaf151-B9] Best O, Ban S. Adolescence: physical changes and neurological development. British Journal of Nursing 2021;30:272–5. 10.12968/bjon.2021.30.5.272 33733842

[daaf151-B10] Białek-Dratwa A, Szczepańska E, Szymańska D et al Neophobia—a natural developmental stage or feeding difficulties for children?. Nutrients 2022;14:1521. 10.3390/nu14071521 35406134 PMC9002550

[daaf151-B11] Binder A, Naderer B, Matthes J. Shaping healthy eating habits in children with persuasive strategies: toward a typology. Front Public Health 2021;9:676127. 10.3389/fpubh.2021.676127 34568250 PMC8455872

[daaf151-B12] Birch LL, Fisher JO. Development of eating behaviors among children and adolescents. Pediatrics 1998;101:539–49. 10.1542/peds.101.S2.539 12224660

[daaf151-B13] Boyland E, Backholer K, Potvin Kent M et al Unhealthy food and beverage marketing to children in the digital age: global research and policy challenges and priorities. Annu Rev Nutr 2024;44:471–97. 10.1146/annurev-nutr-062322-014102 38631811

[daaf151-B14] Bozzola E, Spina G, Agostiniani R et al The use of social media in children and adolescents: scoping review on the potential risks. Int J Environ Res Public Health 2022;19:9960. 10.3390/ijerph19169960 36011593 PMC9407706

[daaf151-B15] Braun V, Clarke V. Thematic Analysis: A Practical Guide. UK: Sage, 2021.

[daaf151-B16] Braun V, Clarke V. Toward good practice in thematic analysis: avoiding common problems and be(com)ing a knowing researcher. Int J Transgend Health 2023;24:1–6. 10.1080/26895269.2022.2129597 36713144 PMC9879167

[daaf151-B17] Braun V, Clarke V. A critical review of the reporting of reflexive thematic analysis in health promotion international. Health Promot Int 2024;39:daae049. 10.1093/heapro/daae049 38805676 PMC11132294

[daaf151-B18] Bronfenbrenner U . The bioecological model from a life course perspective: reflections of a participant observer. In: Moen P, Elder GH Jr, Lüscher K (eds.), Examining Lives in Context: Perspectives on the Ecology of Human Development. USA: American Psychological Association, 1995, 599–618.

[daaf151-B19] Brooks N, Begley A. Adolescent food literacy programmes: a review of the literature. Nutr Diet 2014;71:158–71. 10.1111/1747-0080.12096

[daaf151-B20] Calvert S, Dempsey RC, Povey R. Delivering in-school interventions to improve dietary behaviours amongst 11–16-year-olds: a systematic review. Obes Rev 2019;20:543–53. 10.1111/obr.12797 30550629

[daaf151-B21] Center for Nutrition Policy and Promotion (CNPP) . *MyPlate*, 2023. https://www.fns.usda.gov/cnpp/myplate-miplato (4 December 2025, date last accessed).

[daaf151-B22] Centers for Disease Control and Prevention (CDC) . *National Health and Nutrition Examination Survey Data*, 2021. https://www.cdc.gov/nchs/nhanes/?CDC_AAref_Val=https://www.cdc.gov/nchs/nhanes/index.htm (24 November 2024, date last accessed).

[daaf151-B23] Clark T, Foster L, Bryman A et al Bryman's Social Research Methods, 6th edn. UK: Oxford University Press, 2021.

[daaf151-B24] Collado-Soler R, Alférez-Pastor M, Torres FL et al A systematic review of healthy nutrition intervention programs in kindergarten and primary education. Nutrients 2023;15:541. 10.3390/nu15030541 36771248 PMC9921877

[daaf151-B25] Coulthard H, Harris G, Emmett P. Long-term consequences of early fruit and vegetable feeding practices in the United Kingdom. Public Health Nutr 2010;13:2044–51. 10.1017/S1368980010000790 20529400

[daaf151-B26] Crawford B, Low JY, Newman L. Understanding barriers of eating unfamiliar fruits and vegetables in children using ‘Sensory Play’: a narrative review. Int J Food Sci Technol 2023;58:4075–87. 10.1111/ijfs.16521

[daaf151-B27] Creswell JW, Creswell JD. Research Design: Qualitative, Quantitative, and Mixed Methods Approaches. USA: Sage publications, 2017.

[daaf151-B28] Criss S, Horhota M, Wiles K et al Food cultures and aging: a qualitative study of grandparents’ food perceptions and influence of food choice on younger generations. Public Health Nutr 2020;23:221–30. 10.1017/S1368980019002489 31566158 PMC10200626

[daaf151-B29] Cuykx I, Lochs C, Van Royen K et al What are food media (messages)? A scoping review to clarify food media, food messages and food content in academic writing. Br Food J 2024;126:2746–68. 10.1108/BFJ-05-2023-0382

[daaf151-B30] Department of Health . *The Food Pyramid. Health Service Executive*, 2016. https://assets.hse.ie/media/documents/food-pyramid-simpleversion.pdf (5 December 2024, date last accessed)..

[daaf151-B31] Dixon H, Niven P, Scully M et al Food marketing with movie character toys: effects on young children's preferences for unhealthy and healthier fast food meals. Appetite 2017;117:342–50. 10.1016/j.appet.2017.07.014 28712977

[daaf151-B32] Ghaljaie F, Naderifar M, Goli H. Snowball sampling: a purposeful method of sampling in qualitative research. Strides Dev Med Educ 2017;14:1–6. 10.5812/sdme.67670

[daaf151-B33] Ghosh D, Khan IA, Yadav S et al The impact of early childhood nutrition on long-term health outcomes: a prospective cohort study. J Popul Ther Clin Pharmacol 2024;31:317–24. 10.53555/jptcp.v31i3.4789

[daaf151-B34] Golafshani N . Understanding reliability and validity in qualitative research. Qual Rep 2003;8:597–607.

[daaf151-B35] Harris JL, Fleming-Milici F, Gearhardt AN et al Digital food marketing and children’s health and well-being. In: Handbook of Children and Screens: Digital Media, Development, and Well-Being from Birth Through Adolescence. Cham: Springer Nature Switzerland, 2025, 81–90.

[daaf151-B36] Hawkins A, Rundle R. School food hero and the battle of the food foes: a story of public health policy, power imbalance and potential. Soc Sci Med 2024;342:116520. 10.1016/j.socscimed.2023.116520 38232532

[daaf151-B37] Hayward K . Bordering on Brexit: Views from Local Communities in the Central Border Region of Ireland/Northern Ireland. Northern Ireland: Queen's University Belfast, 2017.

[daaf151-B38] Herle M, De Stavola B, Hübel C et al A longitudinal study of eating behaviours in childhood and later eating disorder behaviours and diagnoses. Br J Psychiatry 2020;216:113–9. 10.1192/bjp.2019.174 31378207 PMC7000294

[daaf151-B39] Hoffner CA, Bond BJ. Parasocial relationships, social media, & well-being. Curr Opin Psychol 2022;45:101306. 35219157 10.1016/j.copsyc.2022.101306

[daaf151-B40] Holloway TP, Dalton L, Hughes R et al School gardening and health and well-being of school-aged children: a realist synthesis. Nutrients 2023;15:1190. 10.3390/nu15051190 36904189 PMC10005652

[daaf151-B41] Hollywood L, Issartel J, Gaul D et al Cook like a Boss Online: an adapted intervention during the COVID-19 pandemic that effectively improved children’s perceived cooking competence, movement competence and wellbeing. Int J Behav Nutr Phys Act 2022;19:146. 10.1186/s12966-022-01378-x 36494840 PMC9733269

[daaf151-B42] Jongenelis MI, Morley B, Worrall C et al Grandparents’ perceptions of the barriers and strategies to providing their grandchildren with a healthy diet: a qualitative study. Appetite 2021;159:105061. 10.1016/j.appet.2020.105061 33271201

[daaf151-B43] Kemmer D . Tradition and change in domestic roles and food preparation. Sociology 2000;34:323–33. 10.1177/S0038038500000201

[daaf151-B44] Lambert SD, Loiselle CG. Combining individual interviews and focus groups to enhance data richness. J Adv Nurs 2008;62:228–37. 10.1111/j.1365-2648.2007.04559.x 18394035

[daaf151-B45] Lavelle F . A critical review of children's culinary nutrition interventions, the methodologies used and their impact on dietary, psychosocial and wellbeing outcomes. Nutr Bull 2023;48:6–27. 10.1111/nbu.12596 36377697

[daaf151-B46] Lutfeali S, Ward T, Greene T et al Understanding the extent of adolescents’ willingness to engage with food and beverage companies’ Instagram accounts: experimental survey study. JMIR Public Health Surveill 2020;6:e20336. 10.2196/20336 33107836 PMC7655467

[daaf151-B47] Maher J, Supski S, Wright J et al Children, ‘healthy’ food, school and family: the ‘[n]ot really’ outcome of school food messages. Child Geogr 2020;18:81–95. 10.1080/14733285.2019.1598546

[daaf151-B48] Maksi SJ, Keller KL, Dardis F et al The food and beverage cues in digital marketing model: special considerations of social media, gaming, and livestreaming environments for food marketing and eating behavior research. Front Nutr 2024;10:1325265. 10.3389/fnut.2023.1325265 38384857 PMC10880034

[daaf151-B49] McCrickerd K . Cultivating self-regulatory eating behaviours during childhood: the evidence and opportunities. Nutr Bull 2018;43:388–99. 10.1111/nbu.12355

[daaf151-B50] McGuffin LE, Price RK, McCaffrey TA et al Parent and child perspectives on family out-of-home eating: a qualitative analysis. Public Health Nutr 2015;18:100–11. 10.1017/S1368980014001384 25100625 PMC10271375

[daaf151-B51] McKinsey & Company . *Ordering in: The Rapid Evolution of Food Delivery*, 2021. https://www.mckinsey.com/industries/technology-media-and-telecommunications/our-insights/ordering-in-the-rapid-evolution-of-food-delivery (5 December 2024, date last accessed).

[daaf151-B52] Meléndez-Illanes L, González-Díaz C, Álvarez-Dardet C. Advertising of foods and beverages in social media aimed at children: high exposure and low control. BMC Public Health 2022;22:1795. 10.1186/s12889-022-14196-4 36138364 PMC9494888

[daaf151-B53] Mikkelsen MV, Husby S, Skov LR et al A systematic review of types of healthy eating interventions in preschools. Nutr J 2014;13:56. 10.1186/1475-2891-13-56 24906305 PMC4074866

[daaf151-B54] Morrill BA, Madden GJ, Wengreen HJ et al A randomized controlled trial of the food dudes program: tangible rewards are more effective than social rewards for increasing short-and long-term fruit and vegetable consumption. J Acad Nutr Diet 2016;116:618–29. 10.1016/j.jand.2015.07.001 26297598

[daaf151-B55] National Health Service . *Eat Well Guide*, 2022. https://www.nhs.uk/live-well/eat-well/food-guidelines-and-food-labels/the-eatwell-guide/ (5 December 2024, date last accessed).

[daaf151-B56] Nieto C, Espinosa F, Valero-Morales I et al Digital food and beverage marketing appealing to children and adolescents: an emerging challenge in Mexico. Pediatr Obes 2023;18:e13036. 10.1111/ijpo.13036 37078451

[daaf151-B57] Papalia DE, Feldman RD. A Child’s World: Infancy Through Adolescence, 12th edn. USA: McGraw-Hill, 2011.

[daaf151-B58] Pastore M, Indrio F, Bali D et al Alarming increase of eating disorders in children and adolescents. J Pediatr 2023;263:113733. 10.1016/j.jpeds.2023.113733 37717906

[daaf151-B59] Perdew M, Liu S, Naylor PJ. Family-based nutrition interventions for obesity prevention among school-aged children: a systematic review. Transl Behav Med 2021;11:709–23. 10.1093/tbm/ibaa082 32893869

[daaf151-B60] Powell F, Herle M, De Stavola B et al Non-responsive feeding practices and their impact on children's eating behaviors. Appetite 2023;168:105731. 10.1016/j.appet.2023.105731

[daaf151-B61] Prior MT . Accomplishing rapport in qualitative research interviews: empathic moments in interaction. Appl Linguist Rev 2018;9:487–511. 10.1515/applirev-2017-0029

[daaf151-B62] Public Health England . *NDNS: results from years 9 to 11 (2016 to 2017 and 2018 to 2019)*, 2020. https://www.gov.uk/government/statistics/ndns-results-from-years-9-to-11-2016-to-2017-and-2018-to-2019/ndns-results-from-years-9-to-11-combined-statistical-summary 9 (14 January 2025, date last accessed).

[daaf151-B63] Qutteina Y, Hallez L, Raedschelders M et al Food for teens: how social media is associated with adolescent eating outcomes. Public Health Nutr 2021;24:4156–65. 10.1017/S1368980020003079 34325764 PMC8883778

[daaf151-B64] Reddy G, van Dam RM. Food, culture, and identity in multicultural societies: insights from Singapore. Appetite 2020;149:104633. 10.1016/j.appet.2020.104633 32084519

[daaf151-B65] Russell CG, Worsley A, Campbell KJ et al Engaging children in developing interventions to improve eating behaviors: a participatory research approach. Int J Behav Nutr Phys Act 2023;20:Article 47. 10.1186/s12966-023-01407-3

[daaf151-B66] Ruxton CH, Ruani MA, Evans CE. Promoting and disseminating consistent and effective nutrition messages: challenges and opportunities. Proc Nutr Soc 2023;82:394–405. 10.1017/S0029665123000022 36603858

[daaf151-B67] Sacks G, Looi E. The advertising policies of Major social Media platforms overlook the imperative to restrict the exposure of children and adolescents to the promotion of unhealthy foods and beverages. Int J Environ Res Public Health 2020;17:4172. 10.3390/ijerph17114172 32545343 PMC7312784

[daaf151-B68] Slaughter CW, Bryant AH. Hungry for love: the feeding relationship in the psychological development of young children. Perm J 2004;8:23. 10.7812/TPP/03-063 26704602 PMC4690704

[daaf151-B69] Smith R, Kelly B, Yeatman H et al Food marketing influences children's attitudes, preferences and consumption: a systematic critical review. Nutrients 2019;11:875. 10.3390/nu11040875 31003489 PMC6520952

[daaf151-B70] Stavitz J . Understanding micronutrient access through the Lens of the social ecological model: exploring the influence of socioeconomic factors—a qualitative exploration. Nutrients 2024;16:1757. 10.3390/nu16111757 38892693 PMC11174576

[daaf151-B71] Tatlow-Golden M, Garde A. Digital food marketing to children: exploitation, surveillance and rights violations’. Glob Food Sec 2020;27:100423. 10.1016/j.gfs.2020.100423

[daaf151-B72] Théodore FL, López-Santiago M, Cruz-Casarrubias C et al Digital marketing of products with poor nutritional quality: a Major threat for children and adolescents. Public Health 2021;198:263–9. 10.1016/j.puhe.2021.07.040 34492506

[daaf151-B73] Thomas J, Sutcliffe K, Harden A et al Children and healthy eating: a systematic review of barriers and facilitators. Health Educ Res 2003;18:191–206. 10.1093/her/18.2.191 12729178

[daaf151-B74] United Nations . *Convention on the Rights of the Child*, 1989. https://www.ohchr.org/en/instruments-mechanisms/instruments/convention-rights-child Say something (14 January 2025, date last accessed).

[daaf151-B75] van der Bend DL, Beunke TA, Shrewsbury VA et al My feed is what I eat? A qualitative study on adolescents’ awareness and appreciation of food marketing on social media. J Hum Nutr Diet 2024;37:1320–35. 10.1111/jhn.13336 38856698

[daaf151-B77] Varela P, De Rosso S, Moura AF et al Bringing down barriers to children’s healthy eating: a critical review of opportunities, within a complex food system. Nutr Res Rev 2024;37:331–51. 10.1017/S0954422423000203 37746804

[daaf151-B78] VegPower . *Eat Them to Defeat Them: Evaluation report 2022*, 2022. https://vegpower.org.uk/wp-content/uploads/2022/09/Eat-Them-to-Defeat-Them-Evakuation-Report-2022.pdf (14 January 2025, date last accessed).

[daaf151-B79] Wolfson JA, Leung CW. Food insecurity and COVID-19: disparities in early effects for US adults. Nutrients 2020;12:1648. 32498323 10.3390/nu12061648 PMC7352694

[daaf151-B80] World Health Organization (WHO) . *Report of the Commission on Ending Childhood Obesity*, 2016. https://apps.who.int/iris/handle/10665/204176 (14 January 2025, date last accessed).

[daaf151-B81] World Health Organization (WHO) . *Infant and Young Child Feeding*, 2023. https://www.who.int/news-room/fact-sheets/detail/infant-and-young-child-feeding (14 January 2025, date last accessed)

[daaf151-B82] Wright-Pedersen S, Vidgen H, Gallegos D. Children's descriptions of their involvement within everyday food practices’. Appetite 2024;200:107517. 10.1016/j.appet.2024.107517 38815691

[daaf151-B83] Wyllie J, Baxter S, Kulczynski A. Healthy kids: examining the effect of message framing and polarity on children's attitudes and behavioral intentions. J Advert 2015;44:140–50. 10.1080/00913367.2015.1018462

